# Dietary elimination of children with food protein induced gastrointestinal allergy – micronutrient adequacy with and without a hypoallergenic formula?

**DOI:** 10.1186/2045-7022-4-31

**Published:** 2014-10-03

**Authors:** Rosan Meyer, Claire De Koker, Robert Dziubak, Heather Godwin, Gloria Dominguez-Ortega, Neil Shah

**Affiliations:** Gastroenterology Department, Great Ormond Street Hospital for Children NHS foundation Trust, London, UK; Department of Nutrition and Dietetics, Chelsea and Westminster Hospital NHS Foundation Trust, London, UK; Katholic University Hospital, TARGID, Leuven, Belgium; Gastroenterology and Nutrition Department, Niño Jesús Children University Hospital, Madrid, Spain

## Abstract

**Background:**

The cornerstone for management of Food protein-induced gastrointestinal allergy (FPGIA) is dietary exclusion; however the micronutrient intake of this population has been poorly studied. We set out to determine the dietary intake of children on an elimination diet for this food allergy and hypothesised that the type of elimination diet and the presence of a hypoallergenic formula (HF) significantly impacts on micronutrient intake.

**Method:**

A prospective observational study was conducted on children diagnosed with FPIGA on an exclusion diet who completed a 3 day semi-quantitative food diary 4 weeks after commencing the diet. Nutritional intake where HF was used was compared to those without HF, with or without a vitamin and mineral supplement (VMS).

**Results:**

One-hundred-and-five food diaries were included in the data analysis: 70 boys (66.7%) with median age of 21.8 months [IQR: 10 - 67.7]. Fifty-three children (50.5%) consumed a HF and the volume of consumption was correlated to micronutrient intake. Significantly (p <0.05) more children reached their micronutrient requirements if a HF was consumed. In those without a HF, some continued not to achieve requirements in particular for vitamin D and zinc, in spite of VMS.

**Conclusion:**

This study points towards the important micronutrient contribution of a HF in children with FPIGA. Children, who are not on a HF and without a VMS, are at increased risk of low intakes in particular vitamin D and zinc. Further studies need to be performed, to assess whether dietary intake translates into actual biological deficiencies.

## Background

Food allergy is an immune-mediated reaction which can be either antibody-driven (IgE-mediated) or cell-mediated (non-IgE-mediated), and elicits reactions which are reproducible upon re-exposure to the specific food [1]. The prevalence of food allergy ranges from 2.5%-6% in children, depending on age, with the most common causative foods being cow’s milk, hen’s egg, soya bean, wheat, fish, shellfish, peanuts and tree nuts [2–4]. Food protein-induced gastrointestinal allergy (FPIGA), which include food protein induced enterocolitis, proctocolitis, enteropathy and food protein induced gastro-oesophageal reflux are classified as non-IgE mediated according to Johansson et al. [1]. The diagnosis in clinical practice for these allergic conditions is made by an elimination diet followed by a re-challenge of the offending allergen [5, 6]. Guidelines have acknowledged that the period of elimination for patients presenting with FPIGA may be longer before a diagnosis can be confirmed, related to both the severity of gastrointestinal symptoms and whether the correct causative foods have been eliminated [7]. As a result this type of food allergy, often requires the elimination of multiple foods at once and for longer periods to gain symptom control [8].

The process of dietary elimination has been shown to predispose children to nutritional inadequacies, in particular nutrient deficiencies have been documented in cow’s milk protein allergy (CMPA), with growth faltering, vitamin D and calcium deficiency commonly reported [9, 10]. Isolauri et al. [11] found that 34–45% of energy and 35–47% of protein intake is provided by hypoallergenic formula (HF), with 91% of children with CMPA who consumed sufficient volumes of HF meeting the recommended intake for micronutrients, in particular for calcium and vitamin D. The prevention of suboptimal nutrient intake is extremely important to avert long term stunting and other health complications associated with vitamin and mineral deficiencies [11–13]. Although current guidelines suggest continuing HF until the age of 2 years [14, 15], many children above the age of 1 are switched to alternative over-the-counter preparations, including oat, rice and coconut milk, often due to taste preferences and cost restraints related to local healthcare. Although cases of micronutrient deficiency and under-nutrition have been reported with these milk preparations, it is generally assumed that children achieve micronutrient requirements if a balanced dietary intake as assessed by dietitian and vitamin and mineral supplementation (VMS) is prescribed as required [16].

Studies investigating dietary adequacy of elimination diets have focused mainly on IgE-mediated food allergy, with paucity of data on the impact on the elimination diet in non-IgE mediated FPIGA. We therefore set out to determine the dietary intake of children who require an elimination diet for FPIGA and hypothesised that the type of elimination and the presence of a HF significantly impact on micronutrient adequacy.

## Methods

### Subjects and study design

This was a prospective, observational study performed in the gastroenterology department, at Great Ormond Street Hospital for Children NHS Foundation Trust, London, United Kingdom (UK). Ethical approval (number 11/LO/1177) was obtained for this study. Parents of children aged 4 weeks – 16 years without non-allergic co-morbidities who were required to follow an elimination diet for the diagnosis of suspected FPIGA, were eligible to take part in the study. The inclusion of children in this study depended on the improvement of symptoms following an elimination diet: a Likert Scale gastro-intestinal symptom questionnaire [17] that has previously been developed by the same research team and published, was administered at baseline prior to commencing the elimination diet and again at 4 and 8 weeks after commencing the dietary elimination. Only children that improved in their score (i.e. symptoms improved) were enrolled in the study. Dietary advice was given by the hospital dietitians following the diagnosis of suspected FPIGA and diet sheets from the Food Allergy Specialist Group of the UK British Dietetic Association where available or local diet sheets were used for the consultations. The recommendations of VMS were determined on an individual basis, using mainly prescribable products available on the National Health Service in the UK.

### Dietary intake

A 3-day estimated food diary (as described by Lee & Nieman) [18] was recorded a minimum of 4 weeks after initiating the exclusion diet. All patients and parents included in this study saw the same research dietitian and were given detailed instructions on how to complete the diary as accurately as possible, including a portion size guide and a sample menu. HF consumption (including type and volume), milk alternatives for older children and VMS were also documented daily. Although we did ask parents to document whether their child was breastfed or not and to include their breastfeeding routine in the food diary, we were not able to link the time of breast feeding to a volume of consumption in the individual child. All infants that were exclusively breastfed or received ≥ 2 breast feeds per day in addition to their HF were therefore excluded from the dietary analysis.

Food diaries were discussed with parents and any unclear entries were clarified by the researcher at the time of research appointment or through telephone communication after the appointment. The UK Food Portion Sizes published by the Food Standard Agency was used to help guide parents and healthcare professionals in estimating the correct portion size whenever portions needed converting from household measures to grams [19].

Nutritional intake data was assessed using Dietplan 6 Software (Forestfield Software Limited, UK). Any foods, in particular specialist foods free from allergens, as well as HF and VMS not available on the software database were manually added by the researcher, and product information was obtained from the manufacturer where necessary. Children who were prescribed VMS had two versions of food diary analysed: including and excluding VMS.

Dietary intakes for each child were compared to the age appropriate UK Dietary Reference Values and adequacy of intake was defined as follows: inadequate intake was expressed as achieving less than the Lower Reference Nutrient Intake (LRNI – meeting nutrient requirements for 2.5% of population) and adequate intake as achieving the Reference Nutrient Intake (RNI – meeting nutrient requirements for 97.5% of population), with consumption between LRNI and excessive intake still classified as “adequate intake” [20]. Where LRNI was not available we used a cut-off of less than 67% of the RNI, as outlined by the Scientific Advisory Committee on Nutrition in the UK [21]. As Safe Upper Limits in the UK have been calculated on an assumed body mass of 60 kg and there is paucity of data for on excessive micronutrient intakes in many of the micronutrients for children, we arbitrarily defined excessive intake as more than 200% of the RNI [22].

### Statistical analysis

Statistical analysis was performed using IBM SPSS Statistics for Windows, version 21 (Armonk, NY). Continuous variables were presented as medians with interquartile ranges and categorical variables are presented as frequencies. The Chi-square test was used to compare differences in proportions of micronutrient intake of children with or without a HF as well as differences in those with/without a VMS. To compare the ages between the above two groups the Mann-Whitney U test was used. Spearman test was used to check correlation between volume of formula intake and Reference Nutrient Intake (%RNI). All tests were two-tailed and significance level was set to 0.05.

Multivariate regression analysis was used to ascertain what factors influence nutrient intake.

We stratified our cohort into two groups depending on the consumption of a HF, and developed separate regression models for each group. Factors included in the regression analysis were chosen based on the outcome of univariate analysis. For both groups the following factors were considered as independent variables: gastrointestinal symptoms, feeding difficulties, number of foods excluded (1, 2, 3, 4+ foods excluded), type of food excluded (wheat, egg, soya, milk), age (months) and gender. We also considered over-the-counter alternative milks as a factor in children without a HF. As all children included for this analysis had a CMPA, we did not include this as an independent variable. For those who received HF we also included volume of HF (ml) as a variable. For this analysis, food diaries without VMS supplementation were used for all children as our primary objective was to assess the impact of HF versus no HF on dietary intake and VMS would have biased the analysis, however the impact of VMS was assessed separately as described above. Logarithmic transformation was used for non-normally distributed data.

## Results

### Subjects

**Figure 1:**
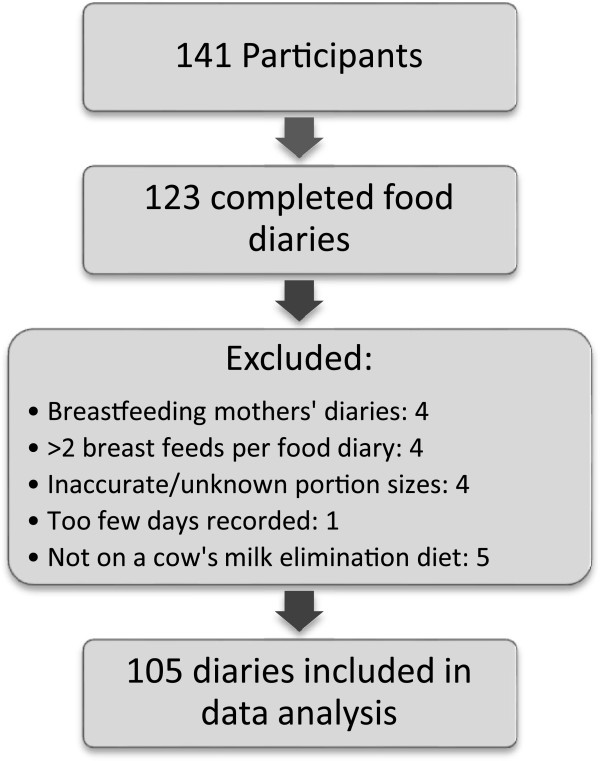
**Number of participants that completed the study and useable food diaries.**

**Figure 2:**
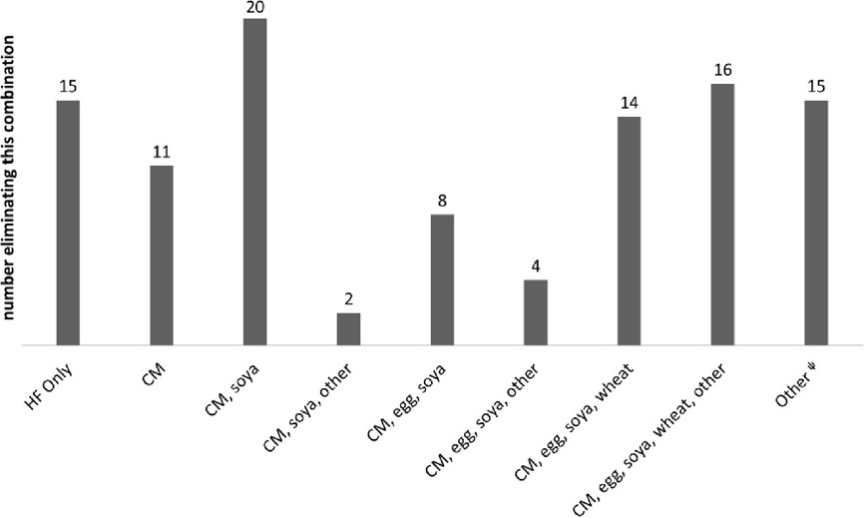
**Combinations of foods excluded.** (HF: hypoallergenic formula, CM: cow’s milk). ^ψ^Other combinations, 2+ foods excluded, milk excluded in all cases.

Of the 141 patients recruited according to our inclusion criteria, 123 completed the food diaries, but only 105 food diaries were included in the data analysis due to some infants being breast fed, inadequate information on portion sizes (Figure 1) and 5 children did not require a cow’s milk elimination diet. Diaries were obtained from 70 boys (66.7%) and the median age of the cohort was 21.8 months [IQR: 10 to 67.7]. In this study, we had 15 (13.3%) children on HF only, 11/105 (10.5) eliminated 1 food, 24/105 (22.9%) two foods, 18/105 (17.1%) three foods and 37/105 (35.2%) four and more foods. Elimination diets are depicted in Figure 2, which indicates that the most common combination of dietary elimination was cow's milk and soya as well as cow's milk, soya, egg and wheat/others.

### Nutrient intake with/without a HF and VMS

From our cohort 53 children (50.5%) consumed a HF, of which 50 (94.3%) consumed an amino acid formula (AAF) and 3 (5.7%) an extensively hydrolysed formula (EHF). The median volume of formula consumption was 664.4 ml per day, [IQR: 419 - 839]. As expected, children consuming HF were significantly younger than those not consuming an HF (median 10 months [IQR: 6.2 to 16.7] vs. 66.3 months [IQR: 30.1 - 112.9], p < 0.0001).

**Figure 3:**
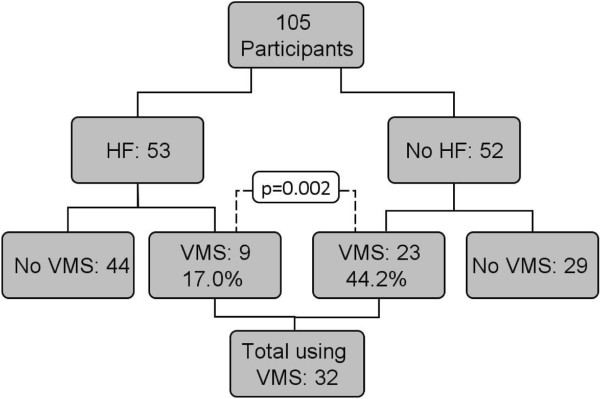
**Number of children with/without HF and with/without VMS.** Statistical significance was found between the proportion of children with and without a VMS. HF: hypoallergenic formula; VMS: micronutrient supplementation.

Thirty-two (30.5%) children of the total cohort received a VMS. Significantly more children received a VMS if they were not on the HF (44.2% (23/52) vs. 17% (9/53), p = 0.007). Figure 3 provides an overview on the consumption of VMS in children on a HF or without a HF who were consuming over-the-counter calcium enriched preparations (17 oat milk, 14 rice milk, 9 coconut milk, 7 almond milk and 1 hazelnut milk). A wide variety of different VMS were used, which included multivitamins (7 children, 21.9%), multivitamin- and mineral supplements (17 children, 53.1%), and single nutrient supplements such as calcium, vitamin D or iron (8 children, 25%).

We analysed the proportions of children that did not achieve their LRNI with or without a HF and assessed the impact of a VMS in these two groups (Table 1). The data showed that without any VMS, children with a HF were more likely to achieve their LRNI for all micronutrients (Table 1 Column A and B) and differences between these groups were significant except for riboflavin and iron. Without a VMS, 5.7% of children with a HF versus 28.8% without a HF did not achieve their LRNI for zinc, similarly 22.6% (HF group) and 94,2% (without HF) did not achieve the LRNI for vitamin D. We also compared micronutrient intake for those children in the two groups who received supplementation (Table 1 Column C and D) and found that after the addition of the VMS, except for vitamin D (2 children) and calcium (1 child) in the group receiving no HF, all children achieved their LRNI. Unsurprisingly the volume of HF consumption was positively correlated with micronutrient intake: vitamin D [r = 0.900, p < 0.001], zinc [r = 0.854, p < 0.001], vitamin A [r = 0.424, p = 0.002], riboflavin [r = 0.595, p < 0.001], iron [r = 0.617, p < 0.001], copper [r = 0.609, p = p < 0.001], selenium [r = 0.375, p = 0.006]. Only the intake of calcium was not correlated with volume of HF [r = 0.123, p = 0.381].

**Table 1:** **Proportion children with or without a HF and VMS that do not achieve their LRNI (shaded areas in columns indicate no significant difference)**

	Dietary intake without VMS				Dietary intake of children that received VMS			
Nutrient	Column A	Column B	P value	Total % not achieving LRNI	Column C	Column D	P value	Total % not achieving LRNI
	No HF	With HF			No HF	With HF		
	n/total	n/total			n/total	n/total		
	(%)	(%)			(%)	(%)		
Zinc	15/52 (28.8)	3/53 (5.7)	0.002	17.1	0/5 (0)	0/2 (0)	0.308	0
Vitamin D	49/52 (94.2)	12/53 (22.6)	0.000	58.1	2/6 (33.3)	0/7 (0)	0.046	15.4
Vitamin A	8/52 (15.4)	1/53 (1.9)	0.016	8.6	0/14 (0)	0/7 (0)	0.095	0
Selenium	8/52 (15.4)	1/53 (1.9)	0.016	8.6	0/3 (0)	--	--	0
Riboflavin	5/52 (9.6)	1/53 (1.9)	0.113	5.7	0/6 (0)	0/9 (0)	0.319	0
Iron	4/52 (12.3)	1/53 (1.9)	0.205	4.8	0/6 (0)	0/3 (0)	1	0
Calcium	8/52 (15.4)	1/53 (1.9)	0.016	8.6	1/15 (6.7)	0/2 (0)	0.662	6%
Copper	7/52 (13.5)	1/53 (1.9)	0.031	7.6	0/3 (0)	0/2 (0)	1	0

As current guidance suggests the continuation of HF until two years of age, [15] we also assessed the number of children in our cohort that were still on a HF at that age and its impact on micronutrient intake. In this study 85.7% of the children ≤ 2 years of age had HF versus 9.3% of older children (p < 0.0001). We also assessed the prevalence of any deficient micronutrient intake for the 54 children ≤ 2 years with and without a HF, and found that 17% (8/47) had a deficient intake with versus 74.4% (5/7) (p = 0.006) without the HF.

We performed regression analysis on the stratified cohort (consuming or not consuming HF) using the food diary for all children without VMA to determine which factors were likely to contribute to the intake of particular micronutrients. The univariate analysis found that gastrointestinal symptoms, number of foods excluded (1, 2, 3, 4+ foods excluded), particular food excluded (wheat, egg, soya), age (months) and gender significantly impacted on micronutrient intake for both models and over-the-counter alternative milks for the cohort that did not receive a HF. We excluded gastrointestinal symptoms as a factor, as no previous data indicated these as being of clinical relevance.

Table 2a and b presents the regression models for the cohort with and without a HF. In the regression model for children consuming a HF, volume of HF positively affected nutrient intake for all nutrients except of calcium. Boys on average had higher intake of zinc, calcium, copper, selenium and riboflavin. In the regression model of the cohort not on a HF, age was negatively correlated with nutrient intake of calcium, copper, vitamin D and riboflavin. Those who were on a wheat elimination diet on average had a lower intake of zinc, copper, selenium and iron. Boys on average had higher intake of vitamin A and those who had alternative milk in their diets had on average higher intake of iron. Number of excluded foods did not impact on any nutrient intake.

**Table 2:** **a and b: Regression analysis of nutrients intakes for group of consuming HF (Top) and not consuming HF (Bottom)**

a) Subgroup consuming hypoallergenic formula								
	Zinc	Calcium	Copper	Selenium	Vitamin D	Vitamin Aφ	Riboflavinφ	Ironφ
Constant	25.3	100.93	76.42	72.71	19.38	103	106	84.11
HF Volume	0.14***		0.11***	0.07*	0.14***	0.08**	0.05**	0.09***
Male	17.54**	25.56*	21.03*	45.56**			19*	
R^2^	77%	9%	48%	19%	77%	15%	25%	36%
**b) Subgroup without hypoallergenic formula**								
	Zinc	Calcium	Copper	Selenium	Vitamin Dφ	Vitamin Aφ	Riboflavinφ	Ironφ
Constant	95.03	194.49	189.79	137.14	32.23	82.75	191.01	93.57
Age (months)		-0.91***	-0.45**		- 0.73**		- 0.64***	
Wheat exclusion	-38.75***		-58.12***	-53.34**				- 39.7%***
Male						58.0*		
Over-the-counter milk								37.4*
R^2^	30%	40%	28%	17%	12%	7%	27%	28%

*< 0.05, **< 0.01, ***< 0.001.

φLog-linear model.

If variable is not stated, no significant impact was found.

## Discussion

Breast milk remains the gold standard nutritional source for infants with food allergy [15], although methods exist to estimate intake on a population basis, these are not sufficiently accurate in individuals/small groups of patients. This study therefore focused on the dietary adequacy of those children with presumed FPIGA on an elimination diet and the impact of this diet with or without a HF. Although the nutritional benefits of adequate consumption of HF as part of elimination diet in the young IgE mediated allergic child has been highlighted in previous studies in children with [10, 11, 23], no studies to date have evaluated the impact of the elimination diet in non-IgE mediated FPIGA and if no HF was present. More recent data has indicated that the majority of children will not have outgrown their CMPA by 1 year of life [24, 25]. As a result more children will therefore either continue on a HF or progress to an alternative. Our hypothesis that the presence of a HF significantly impacts on micronutrient intake is supported by our findings, moreover we have found that if a child is not on a HF and without a VMS that they are at an increased risk of deficient micronutrient intake (Table 1).

Current national and international guidelines suggest that HF should be continued until the age of 2 years [14, 15, 26]. However this suggestion has mainly been based on consensus rather than evidence of nutritional deficiencies. Although we have only small numbers of children ≤ 2 years of age who are not on a HF, we have demonstrated that if not on a HF that the likelihood of deficient intake is significantly increased. Although this needs to be investigated further in studies developed specifically to capture this data, it seems that current guidance is justified to suggest this as the ideal age for continuing with a HF.

Micronutrients, in particular calcium and vitamin D have been highlighted as deficient in many food allergic children [12, 13]. Dietetic advice often therefore focuses in particular on the intake for these nutrients followed by a suggestion for supplementation. However, micronutrients other than calcium and vitamin D are often not considered. The importance of a HF in terms of micronutrient contribution is not a novel finding in IgE-mediated allergies [11, 23], however to our knowledge this is the first study highlighting this also in non-IgE mediated allergies. We have also shown that children who are not on a HF struggle to achieve the LRNI for the majority of micronutrient intakes, but in particular for vitamin D and zinc. Even with targeted supplementation a small number of children were still not achieving sufficient vitamin D intake. Recent studies have highlighted the link between vitamin D and the development of allergy [27, 28]. Additionally studies have also linked a low serum vitamin D level to an increased severity of eczema [29, 30]. Although there is paucity of data linking a low vitamin D specifically to FPIGA, many publications have focused on its role in inflammatory bowel disease [31, 32]. Whilst the ideal dosage of vitamin D specific for allergic children is still debated, there is irrefutable evidence that vitamin D plays an important immunomodulatory role and that we should at least aim for recommended intake in children with FPIGA, as suggested by current guidelines [14, 15, 27].

Our study found only a low number of children did not achieve the LRNI for calcium, albeit still significantly lower in children without a HF. This is probably not only related to our patients being seen by a paediatric dietitian with knowledge on the increased risk of developing this deficiency, but also associated with the improved calcium content of over-the-counter milk alternatives [33].

Selenium and zinc are important, indispensable trace elements for normal functioning of the human body, especially during early childhood when growth velocity is high. Zinc is involved in protein synthesis, wound healing and tissue maintenance and both are cofactors of antioxidative enzymes [34, 35]. The persistent exposure to allergens can lead to chronic inflammatory changes of the mucosal intestinal lining and increased production of reactive oxidative species, leading to a weakened anti-oxidative barrier [34]. Kamer et al. [34] found that children with both IgE and non-IgE mediated food allergy had lower concentrations of zinc and selenium prior to starting an elimination diet and levels improved following this intervention in comparison to healthy controls. Similarly Ojuwao et al. [36] found that children with allergic colitis had more frequent zinc and selenium deficiencies, indicating these trace elements are not only a concern when it comes to dietary intake in children with FPIGA, but deficiencies are also well documented due to the pathophysiology of this allergy. An Australian expert panel on CMPA highlighted other micronutrients (i.e. iron, folic acid and fat soluble vitamins) as a problem in food protein induced enteropathy, however this particular concern is not mentioned in other guidelines [7, 15, 37]. In addition to the well-described risks related to micronutrient malabsorption in FPIGA, our data clearly indicates that if a food allergic child is not on a HF, that they would not only be at risk of a low intake of vitamin D and calcium, but also zinc and selenium, which are essential trace elements for the immune system.

We also performed regression analysis for children receiving/not receiving a HF to establish which factors impacted on the dietary adequacy of each nutrient. In the regression model for children on a HF, we found that consuming a formula impacted significantly on all micronutrients except for calcium. The fact that calcium intake was not affected by the HF, is likely to be related to most children receiving appropriate dietary advice on how to replace calcium with other calcium rich sources [33].

We were surprised to see that in the regression model without a HF, that the over-the-counter milk alternatives did not impact on calcium intake, which they are enriched with, but rather iron intake was higher. These alternative milks are not enriched with iron, however we hypothesise that iron intake can be higher in this population, as the alternative milks are low in protein and dietary intervention would include increasing the non-cow’s milk protein sources (i.e. meat, fish and pulses), which would automatically increase the iron intake for children in this cohort.

Our hypothesis that the type of dietary elimination impacts on micronutrient intake was only partially proven with only wheat impacting for selected micronutrients. The elimination of wheat seemed to negatively impact on the intake of zinc, copper, selenium and iron in group not consuming HF, however this is most probably related to the dietary analysis of intake. Nutrient content of wheat free products had to be entered manually to the dietary analysis program. In spite of our best efforts we managed to only get macronutrient content (i.e. carbohydrates, protein and fat) and selected micronutrients (i.e. B vitamins, potassium and sodium) for many of these products, which may explain the apparent association with low intake of these trace elements and minerals when wheat is avoided.

In this study the number of foods eliminated did not impact significantly in either groups on the micronutrient intake. We suspect that this may be related to almost all children in our study being under the care of a paediatric dietitian, who would have aimed to provide alternatives of similar nutritional value and ensured nutritional adequacy irrespective of the elimination diet. The support of a paediatric dietitian is essential for optimal dietary management, as suggested by the guidelines on CMPA from European Society for Gastroenterology, Hepatology and Nutrition on the management of CMPA [7].

Finally we also found that being a boy, positively impacted on the intake of zinc, calcium, copper, selenium and riboflavin in children consuming HF, and vitamin A in children not consuming HF. Gender differences have been reported in food choices, with girls eating more fruit and vegetables and boys more meat and protein sources [38, 39]. This may explain the gender differences zinc, copper and iron intake, as protein sources are high in many of these micronutrients, but to a lesser extent for riboflavin and vitamin A. Further studies need to be performed to establish the impact of food preferences in children with food allergies.

The main limitations of the study are related to the fact that we did not recruit a healthy control group to compare micronutrient intake to, and also the assessment of dietary intake through a semi-quantitative food diary. The drawbacks of using this method include motivated responses where parents may change the intake of their child because they are monitored and inaccuracies related to estimated portion sizes [18]. On the other hand this method can give an indication of routine intake and our population was highly motivated to get an accurate assessment for their child’s intake. However, we accept that the ideal would have been not to only rely on dietary intake but use a secondary marker such as blood plasma/urine micronutrient status. This study does also not account for levels of micronutrient absorption which may be affected by having a FPIGA. Jarvinen et al. [40] found increased intestinal permeability in children with IgE-mediated allergy. Although similar data is not available in FPIGA, Ojuawo et al. [36] did find a significant number with trace element deficiencies on elimination diet. Future studies should also aim to investigate the impact of the diagnosis itself on absorption. In addition, we also excluded 8 infants who were breast fed, 4 exclusively and another 4 receiving ≥ 2 breast feeds in addition to solids. Although this may be seen as a limitation of the study, our primary objective was to see the impact of a HF on micronutrient intake and not to assess the nutritional contribution from breast milk. Although methods exist to estimate the volume of breast milk consumption, these are suitable for epidemiological studies and insufficiently accurate for the individual [41].

## Conclusion

This study points towards the important micronutrient contribution of a HF in children with FPIGA. Furthermore, it found that children, who are not on a HF and have not been provided with VMS, are at increased risk of low intakes of many of the micronutrients, in particular vitamin D and zinc. This study also highlights, that current guidance on continuing the HF until 2 years of age is most probably justified and that once a change is made to an over-the-counter milk alternative an individual dietary assessment ideally by a qualified dietitian is indicated, to prevent deficient intake. Nutrient deficiencies have a long lasting impact on growth and development and it is important to achieve these also in food allergic children. However, further studies need to be performed, to assess whether dietary intake in this population translates into actual biological deficiencies.

## Abbreviations

FPIGA: Food protein induced gastrointestinal allergy
IgE: Immunoglobulin E
VMS: Vitamin and mineral supplementation
HF: Hypoallergenic formulas
CMPA: Cow’s milk protein allergy
EHF: Extensively hydrolysed formula
AAF: Amino acid formula.
